# Characterizing Steel Corrosion in Different Alkali-Activated Mortars

**DOI:** 10.3390/ma14237366

**Published:** 2021-11-30

**Authors:** Nina Gartner, Miha Hren, Tadeja Kosec, Andraž Legat

**Affiliations:** Slovenian National Building and Civil Engineering Institute, Dimiceva ul. 12, 1000 Ljubljana, Slovenia; miha.hren@zag.si (M.H.); tadeja.kosec@zag.si (T.K.); andraz.legat@zag.si (A.L.)

**Keywords:** corrosion, alkali-activated mortars, electrochemical impedance spectroscopy, X-ray computed microtomography, visual analysis

## Abstract

Alkali-activated materials (AAMs) present a promising potential alternative to ordinary Portland cement (OPC). The service life of reinforced concrete structures depends greatly on the corrosion resistance of the steel used for reinforcement. Due to the wide range and diverse properties of AAMs, the corrosion processes of steel in these materials is still relatively unknown. Three different alkali-activated mortar mixes, based on fly ash, slag, or metakaolin, were prepared for this research. An ordinary carbon-steel reinforcing bar was installed in each of the mortar mixes. In order to study the corrosion properties of steel in the selected mortars, the specimens were exposed to a saline solution in wet/dry cycles for 17 weeks, and periodic electrochemical impedance spectroscopy (EIS) measurements were performed. The propagation of corrosion damage on the embedded steel bars was followed using X-ray computed microtomography (μXCT). Periodic EIS measurements of the AAMs showed different impedance response in individual AAMs. Moreover, these impedance responses also changed over the time of exposure. Interpretation of the results was based on visual and numerical analysis of the corrosion damages obtained by μXCT, which confirmed corrosion damage of varying type and extent on steel bars embedded in the tested AAMs.

## 1. Introduction

Concrete is the most widely used man-made building material in the world. Its basic components are water, cement, and aggregate. It is estimated that 5–8% of global anthropogenic CO_2_ emissions are released during the production of ordinary Portland cement (OPC), one of the main components of concrete [1,2]. Therefore, one of the major drivers for future innovation is to find new sustainable cementitious materials that have a lower environmental impact. This has led to the development of alternative mineral binders for use in concrete based on the partial or total replacement of conventional OPC clinker with supplementary cementitious materials (SCM). The most commonly used SCMs are industrial by-products with a consistent composition that can be obtained in large and regular quantities, e.g., various slags, fly ash, limestone, and silica fume. Nowadays, however, supplies of these conventional SCMs are also limited; therefore, calcined clays containing kaolinite are considered to be the most promising alternative [3].

Alkali-activated materials (AAMs) use alkali-activated aluminosilicate precursors as a total replacement for OPC and are one of the potential alternatives to binders containing OPC for a wide range of possible applications [4,5]. AAMs are formed by the reaction between various solid aluminosilicate precursors (such as fly ash, slags, and calcined clays) and alkaline activators (e.g., sodium hydroxide and sodium silicate). The product is a hardened binder based on a combination of hydrous alkali-aluminosilicate and/or alkali-alkali earth-aluminosilicate phases [5]. These materials are not considered to be able to totally replace of OPC-based concretes, nor do they offer a one-size-fits-all solution. The wide range of combinations available, however, allows for the formation of materials with various advanced properties, including resistance to acid and heat [6], high strength [7], and radionuclide immobilization [8]. The sustainability of these materials primarily depends on the local availability of the precursors used, as well as selection of the correct type and dose of alkali activator [9].

The use of AAMs for concrete elements also raises the issue of durability, e.g., alkali–silica reaction, resistance to freeze–thaw, chloride-ion penetration, carbonation, corrosion resistance, etc. [10,11]. One of the main remaining issues regarding the use of AAMs in reinforced structures is the understanding of steel corrosion, which is the main cause of structural failure. The primary causes of steel corrosion in OPC concrete are a loss in alkalinity and the ingress of chloride ions [2], which are also considered corrosion initiators in AAMs [12,13,14,15]. The main difference between the corrosion processes in solid porous materials (such as concrete) compared to solutions is the fact that the anodic and cathodic sites are spatially separated, forming a macro cell [16,17], while the distribution of anodic and cathodic sites on a micro scale could also be very important [18,19,20]. At the same time, the porosity of concrete affects the transport of electrolytes and oxygen, which then determines the dynamics of the corrosion processes [21]. Information regarding corrosion rates in concrete should therefore also contain information concerning the type of corrosion, i.e., whether it is placed uniformly across the surface or if it is more localized. Different electrochemical monitoring techniques are commonly used to study corrosion in concrete environments. One such technique used to characterize and quantify the processes of passivation and depassivation in steel used to reinforce concrete is electrochemical impedance spectroscopy (EIS) [22], which is also used to study the corrosion of steel in AAMs [14,23,24]. This non-destructive technique is useful for studying the electrochemical processes of inhomogeneous or multiphase materials. The electrochemical impedance response measured, fitted by the appropriate equivalent circuit model, provides information regarding the electrical properties of the specimen tested, including the resistance and capacitance of the solid and liquid phases, as well as their interfacial properties [24]. However, the pore solution in AAMs can significantly differ from that of OPC-based cements in terms of its chemical, mineralogical, and redox characteristics [25,26,27], which can strongly influence all phases of the corrosion process. This can cause difficulties in the interpretation of parameters obtained by electrochemical techniques usually used for corrosion tests in cementitious materials [26].

Corrosion monitoring techniques generally do not differentiate between general and localized corrosion, and corrosion damage can only be evaluated once the steel has been removed from the concrete through destruction. X-ray computed microtomography (μXCT) has been used in some studies of Portland-cement-based materials over the past two decades [28]. It has also been found to be a useful, non-destructive tool for the visual assessment of steel corrosion in concrete materials, indicating the type, size and location of corrosion damage [29,30,31,32,33,34,35]. In recent years, μXCT has been limited to studying the properties of porosity in alkali-activated materials [36], with this technique not yet used to study the corrosion of steel in these materials.

Following the above referenced literature and state-of-the-art knowledge in the field, it can be concluded that interpretation of electrochemical data in AAMs in relation to corrosion damage and type of corrosion attack is very difficult. Actually, it was not quite clear in specific cases whether an electrochemical response was generated solely by corrosion processes on embedded steel. In this sense, the novelty of our approach is to relate three different types of data (electrochemical parameters, type, and extent of corrosion) to interpret the characteristics of steel corrosion in AAM mixes.

The main aim of this paper was to monitor steel corrosion in AAMs made from different mixes by means of electrochemical impedance spectroscopy (EIS) and to validate the obtained electrochemical parameters. A comprehensive comparison by the type and rate of corrosion obtained by µXCT was performed. An attempt to classify the corrosion damage in the individual AAMs was also made.

## 2. Materials and Methods

The mortar-mix designs used in this research were developed within the scope of RILEM TC 247-DTA, which was aimed at the development of recommendations for testing the durability of alkali-activated materials [13,37,38,39]. Three types of mortar mix were selected, based on three different precursors, i.e., fly ash (FA8), steel slag (S3a-661), and metakaolin (MK2). The exact same raw materials were used as for RILEM TC 247-DTA tests: ground, granulated blast-furnace slag (GGBFS) by ECOCEM^®^ (Fos-sur-Mer, France), hard coal fly ash by BauMineral (Stellenangebote, Germany), and flash-calcined metakaolin by Argeco (AIX EN PROVENCE, France). The original names of the RILEM TC 247-DTA mixes are used in this paper. The exact mix designs are presented in Table 1.

After 105 days, the compressive-strength measurements (according to the standard EN 196-1 [40], test samples’ age was adjusted) of the tested mortars were as follows: MK2 (70 MPa) > S3a-661 (60 MPa) > FA8 (39 MPa). The results of Hg porosimetry after 107 days showed the following proportion of pores in the tested mortars: FA8 (15%) > MK2 (14%) > S3a-661 (11%). The pH values of pore solutions extracted from mortar specimens with a high-pressure device (up to 1000 MPa) [41,42] after 28 days of curing in a humidity chamber (before the exposure) were similar across all the mortars used: pH(FA8) = 12.4, pH(MK2) = 12.5, pH(S3a-661) = 12.8.

Three parallel specimens were prepared for each mortar mix for the corrosion tests. Specimens were cast in 3 cm × 3 cm × 10 cm prism-shaped molds. A cold, ribbed type B 500B reinforcing steel bar (1.0439 [43]) of Φ6 mm diameter was embedded into each specimen. Each end of the steel bar was protected with an epoxy-based coating such that a 9 cm length in the middle was left unprotected and a surface area of 17 cm^2^ was exposed to the mortar. The steel bar was covered with 7 mm of mortar. After casting, the specimens were cured in a humidity chamber for 28 days. Following curing, the specimens were exposed to 3.5% NaCl solution from the pool on the top of each specimen for 17 weeks of wet/dry cycles. Each cycle was one week long, consisting of 3 days wetting, followed by 4 days drying. One week of exposure corresponds to one wet/dry cycle; weeks of exposure are marked as W1 (first week) to W17 (last week).

**Figure 1:**
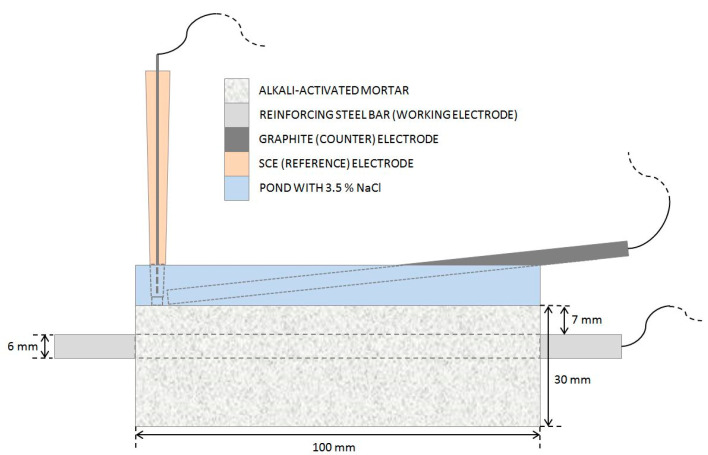
A schematic presentation of the experimental setup used for EIS measurements.

Electrochemical impedance spectroscopy (EIS) measurements (Figure 1) were performed once per cycle, on the last day of the wetting period. The steel reinforcing bar embedded in the mortar specimen was used as the working electrode, while a graphite electrode and a saturated calomel electrode (SCE) submerged in the top pool of the specimen served as the counter and reference electrodes, respectively. The EIS measurements were performed using a Gamry Ref600 potentiostat (Gamry Instruments Inc., Warminster, PA, USA). All potentials refer to the SCE scale. EIS measurements were performed at open-circuit potential (OCP) in the frequency range from 65 kHz to 5 mHz, with 11 points per decade and an amplitude of ±10 mV. The total impedance (|*Z*|_total_) value was estimated as the impedance (|*Z*|) measured at the lowest measured frequency, less the solution resistance (*R*_s_) value.

Before and after the 17-week exposure, analysis of the corrosion damage was performed along the entire exposed steel bar by X-ray computed microtomography (μXCT), using a microXCT-400 (XRadia, Zeiss, Pleasanton, CA, USA). 150 kV source voltage at 5 s exposure time per image, with the obtained voxel resolution of 17 μm, was used. μXCT data were analyzed using Avizo Inspect 2019.1 software (Thermo Fisher Scientific Inc., Waltham, MA, USA). Scans before the exposure were done on steel bars already embedded in AAMs, while μXCT scans at the end of the exposure were done after the steel bars were gently removed from the mortars. Corrosion products and mortar fragments attached to the steel bar were dissolved with cleaning solution HCl (conc.):H_2_O = 50:50 (vol.%) + 3 g/L urotropine solution, and the corrosion damage on the steel bars was visually inspected. In addition to the μXCT analysis, the steel surface was also analyzed by scanning electron microscope (SEM) using JSM-IT500LV (JEOL, Tokyo, Japan) at 20 keV. Visual analysis to determine the type and intensity of corrosion was performed as a complementary method to verify the electrochemical measurements. Final analysis of the chloride content at the depth of the steel reinforcement bar was not possible due to the small size of the specimens and the low mortar cover over the steel bar.

## 3. Results

Electrochemical impedance spectroscopy (EIS) measurements were periodically performed on the steel reinforcement bars embedded in each of the three alkali-activated mortar (AAM) mixes during the wetting/drying cycles (Table 1).

**Figure 2:**
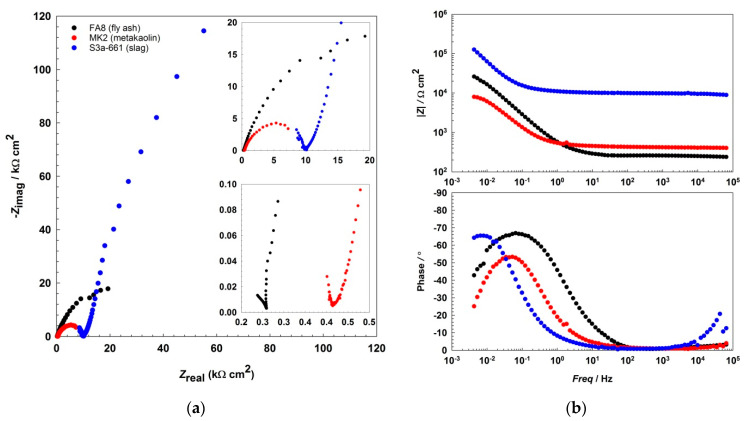
Representative EIS spectra, (**a**) Nyquist, and (**b**) Bode plots recorded during the 1st wetting/drying cycle (W1) in each of the three different alkali-activated mortars.

**Figure 3:**
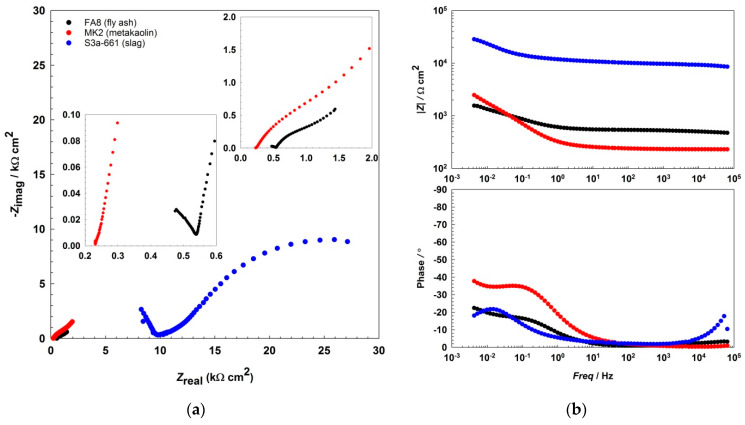
Representative EIS spectra, (**a**) Nyquist, and (**b**) Bode plots recorded during the 8th wetting/ dryingwetting/drying cycle (W8) in each of the three different alkali-activated mortars.

**Figure 4:**
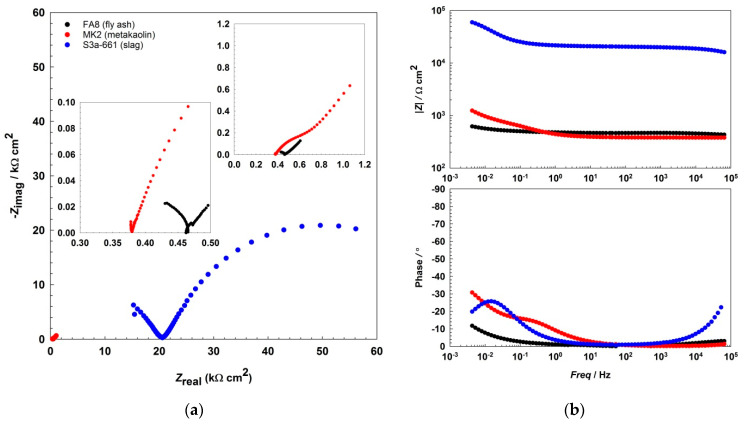
Representative EIS spectra, (**a**) Nyquist, and (**b**) Bode plots recorded during the 17th wetting/drying cycle in each of the three different alkali-activated mortars.

The EIS results show that the impedance responses (Figure 2 and Figure 3, Figure 4) differ between the mortars and also change over the period of exposure. All spectra show an incomplete arc at high frequencies, which is associated with bulk phenomena. The spectra measured in both the fly-ash mortar (FA8) and the metakaolin mortar (MK2) indicate one time constant at the beginning of the exposure (W1, Figure 2), at the point where chlorides were introduced. After several wet/dry cycles of exposure to the chloride solution, an impedance response indicating two time constants became noticeable in these two mortars (FA8 and MK2). The shape measured at W8 (Figure 3) shows two time constants and a straight line at low frequencies, indicating the diffusive properties of the corrosive system investigated, which is more significant at the end of the exposure (W17, Figure 4). The resistance of the exposure media, *R*_s_, in the FA8 mortar slowly rises during the exposure, with the final *R*_s_ value (0.43 kΩ·cm^2^) being 1.8 times higher than its initial value (0.24 kΩ·cm^2^). The resistance (*R*_s_) of the MK2 mortar is similar to that of the FA8 mortar. The spectra measured in the slag mortar (S3a-661) significantly differ from the spectra measured in FA8 and MK2 mortars; the total impedance (|*Z*|_total_) values are much higher, with only one time constant measured, and no diffusive properties of the system were observed. At the end of the exposure, the *R*_s_ value (W17) increases from 10 kΩ·cm^2^ (W1) to 20 kΩ·cm^2^.

**Figure 5:**
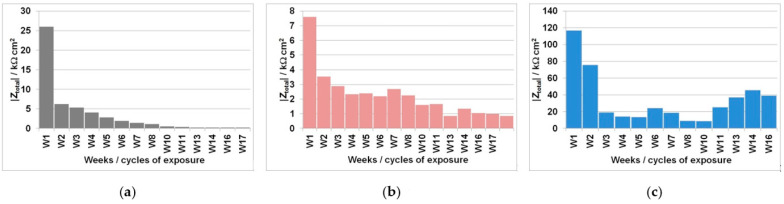
Total impedance (|*Z*|_total_) values periodically measured by EIS during the wetting/drying cycles in the (**a**) FA8, (**b**) MK2, and (**c**) S3a-661 mortars.

The total impedance values (|*Z*|_total_) measured in each wet/dry cycle (W1-W17) for each of the three mortars tested are presented in Figure 5. The *ν*_corr_ values calculated from the EIS parameters measured are presented in Table 2. *j*_corr_ values were calculated using the Stern-Geary equation (Equation (1)) using an estimated constant of *B* = 0.026 V [44]. The |*Z*|_total_ value, less the solution resistance (*R*_s_) value, was used as a near estimation for polarization resistance (*R*_p_). *ν*_corr_ values were calculated according to Equation (2) [22], using an atomic mass value of *AM* = 55.85, a Faraday constant of *F* = 9.65 × 10^4^ As, a valence of *n* = 2, and a steel density of *ρ* = 7.89 g/cm^3^.

(1) $$R_{p}=\frac{B}{j_{\mathrm{corr}}}$$

(2) $$\nu_{\mathrm{corr}}=\frac{AM\cdot j_{\mathrm{corr}}}{n\cdot F\cdot \rho }$$

The corrosion processes intensify as the time of exposure increases, while the total impedance (|*Z*i_ttel_) values decrease over time (Figure 5); corrosion rates (*ν*_corr_) consequently increase over time (Table 2). This is expected as a result of the increase in Cl− concentration in the mortar following each cyclic wetting with the 3.5% NaCl solution. The measured rise in *ν*_corr_ is especially high in the FA8 mortar (Figure 5a), where values gradually increase from 12 μm/year in the 1st wetting/drying cycle to 270 μm/year in the 8th cycle, and finally to 1510 μm/year in the 17th cycle. The equivalent measured increase in *ν*_corr_ values is lower in the MK2 mortar (rising from an initial value of 38 μm/year to a final value of 340 μm/year after 17 cycles) but still significant (Figure 5b). On the contrary, the steel embedded in the S3a-661 mortar does not exhibit significant corrosion rates after 17 cycles of exposure, with the *ν*_corr_ reaching 8 μm/year. However, the |*Z*|_total_ values measured on the steel in the S3a-661 mortar do not decrease evenly (Figure 5c), with the highest corrosion rate (35 μm/year) measured during the 11th week of exposure (W11).

**Figure 6:**
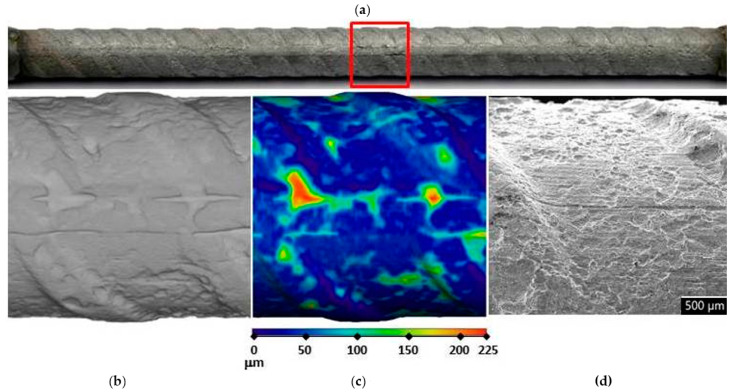
Visual assessment of the steel bar embedded in the FA8 mortar after 17 weeks of exposure; (**a**) photographic image of the steel bar; (**b**,**c**) μXCT images of the most severe corrosion damage; (**d**) SEM image of the representative steel surface (magnitude 35×).

**Figure 7:**
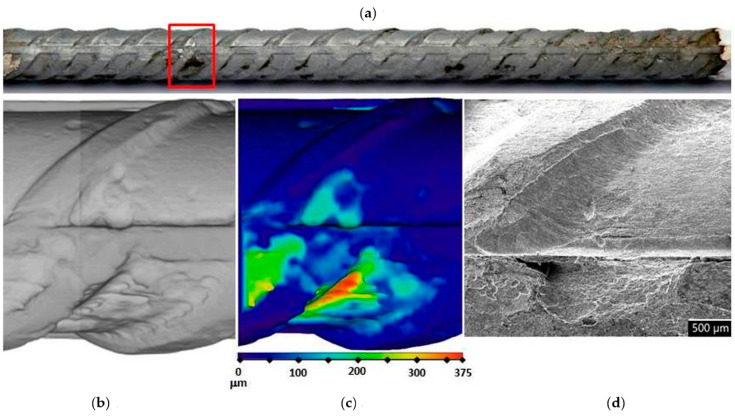
Visual assessment of the steel bar embedded in the MK2 mortar after 17 weeks of exposure; (**a**) photographic image of the steel bar; (**b**,**c**) μXCT images of the most severe corrosion damage; (**d**) SEM image of the representative steel surface (magnitude 35×).

**Figure 8:**
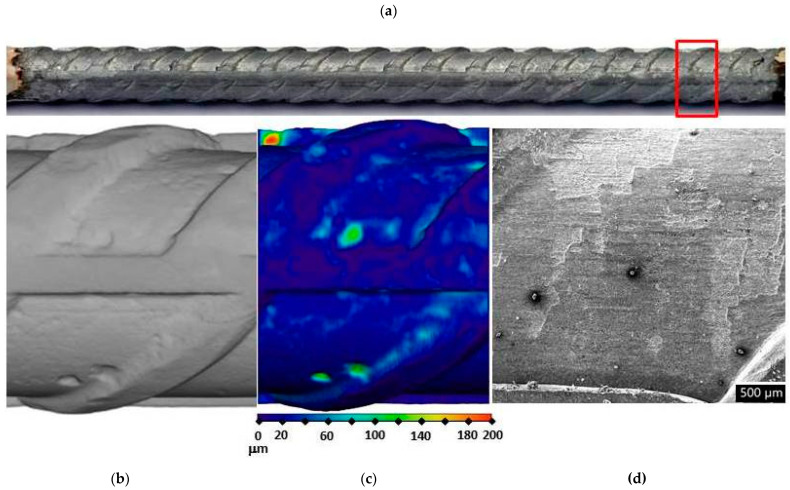
Visual assessment of the steel bar embedded in the S3a-661 mortar after 17 weeks of exposure; (**a**) photographic image of the steel bar; (**b**,**c**) μXCT images of the most severe corrosion damage; (**d**) SEM image of the representative steel surface (magnitude 35×).

In order to assess corrosion damage, visual assessment of the corrosion damage was conducted using photographic analysis, μXCT scans, and SEM imaging. Before the exposure, there was no visible (mechanical) damage on the surface of the steel bars embedded in the mortars. Following the exposure, visual inspection of the steel bars (Figure 6, Figure 7 and Figure 8) confirmed the intensity of the corrosion activity measured by electrochemical impedance spectroscopy (EIS). Images of the steel bar embedded in the FA8 mortar (Figure 6) show severe corrosion damage across the entire surface of the bar. The surface is rough due to the merging of several small local pits, resulting in large areas of corrosion damage spanning the entire surface, with a possible reduction in the rebar diameter. Damage to the steel bar embedded in the MK2 mortar (Figure 7) is fairly deep locally and clearly visible, while the surrounding surface remains undamaged. However, the steel bar embedded in the S3a-661 mortar (Figure 8) shows fairly shallow spots of surface corrosion (Figure 8c), which are mainly limited to areas around the edge.

However, numerical analysis of the visual corrosion damage obtained by μXCT shows a more complex comparison with results obtained using EIS. Corrosion rates (*ν*_corr-EIS_) were calculated from the total impedance (|*Z*|_total_) values measured during each cycle (W1-W17) and then averaged (Table 3). In order to make a comparison, real visual corrosion damage was measured by μXCT (Table 3). The corrosion depths were calculated as the volume of corrosion damage divided by the relevant steel surface. Volume of corrosion damage was obtained directly from the μXCT scans as steel volume before the exposure, less the volume after the exposure. The corrosion rates were calculated from the average (*ν*_corr-A_, Equation (3)) and maximum (*ν*_corr-MAX_, Equation (4)) corrosion depths (Equation (3)). Average corrosion depths (d_A_), maximum corrosion depths (d_MAX_), and time of exposure (t = 119 days) were used for calculation. μXCT analysis confirmed that the average corrosion depths (and consequently, the corrosion rates) are much lower than the maximal local corrosion depths. It is shown that despite the very different types of steel corrosion observed in each mortar, the average damage depths are of the same magnitude (17 μm/year, up to 49 μm/year). The corrosion rates measured by EIS should be comparable to the average corrosion rates measured by μXCT. It can be seen that the corrosion rate of the steel in the FA8 mortar, as measured by EIS (590 μm/year), is higher than the average corrosion rate determined by the μXCT scan (117 μm/year) and lower than the corrosion rate at the point of the deepest damage, as identified by μXCT (690 μm/year). EIS measurements on the MK2 mortar have an even better correlation with the average corrosion rates measured by μXCT, i.e., 185 μm/year vs. 150 μm/year. In both mortars (FA8 and MK2), the corrosion rates measured using EIS were slightly higher than those obtained by μXCT analysis. On the contrary, the corrosion rate of the steel in the S3a-661 mortar measured by EIS (15 μm/year) is lower than the average corrosion rate (54 μm/year) and significantly lower than the highest local corrosion rate obtained by μXCT (613 μm/year). Overall, the corrosion rates measured by EIS correlate well with the average corrosion damage measured by μXCT.

(3) $$\nu_{\mathrm{corr}-A}=\frac{d_{A}}{t}$$

(4) $$\nu_{\mathrm{corr}-\mathrm{MAX}}=\frac{d_{MAX}}{t}$$

## 4. Discussion

This paper presents the results of corrosion measurements obtained from steel installed in three different alkali-activated mortars (AAMs), exposed to saline solution in wet/dry cycles for 17 weeks. In order to quantify and interpret the individual parameters obtained by electrochemical impedance spectroscopy (EIS), the results were compared with final visual analysis by means of X-ray computed microtomography (μXCT) and a scanning electron microscope (SEM).

After 17 weeks of exposure to a chloride solution, through periodical wetting and drying, the types and rates of corrosion were evidently different in each material tested, ranging from very dense but relatively small pits in FA8 (Figure 6) to rather large and deep areas of corrosion with undamaged surrounding areas in MK2, (Figure 7) and to very shallow corrosion spots with only a few small pits in S3a-661 (Figure 8).

In all three AAMs, the electrochemical processes on the steel were measured once per week/cycle by EIS, at the end of each wetting period. Analysis of the impedance spectra shows different impedance responses, depending on the exposure material (i.e., the different mortars) and the time of exposure (i.e., the number of wet/dry cycles), indicating the active/passive state of steel reinforcement and the different types of corrosion mechanisms that should be considered when interpreting the measured EIS values. The incomplete arc at high frequencies that is associated with bulk phenomena [24,45,46,47] is omitted from the interpretation; the discussion focuses on the parts measured at medium and low frequencies, when the system response represents faradaic processes. In the FA8 and MK2 mortars, the spectra obtained show that one time constant was prevalent at the beginning of the exposure. During exposure to chlorides, two time constants are observed, indicating the presence of a double-layer capacitance at the steel/mortar interface in these electrochemical systems [48]. Only one time constant was measured in the S3a-661 mortar. While the diameter of the semicircle in mortars FA8 and MK2 was constantly reducing, in mortar S36a-661, the diameter started to expand in the last few exposure cycles (as can be seen from the |*Z*|_total_ values in Figure 5). Straight lines indicating the Warburg impedance, which represents the resistance to diffusion processes [49] in the mortar, were detected in the low-frequency range of FA8 and MK2 once chlorides had already penetrated into the mortar (W8). The onset of this kind of diffusion impedance indicates that the diffusion of corrosive species into the steel/mortar interface becomes more difficult over time [47], most likely due to the existing presence of corrosion products. A Warburg impedance was not measured at the beginning of the exposure, nor was it measured on the steel embedded in the S3a-661 mortar at any point over the entire period of exposure. This indicates that corrosion products were not yet formed during the initial measurements in mortars FA8 and MK2 and did not form in the S3a-661 mortar throughout the entire period of exposure.

It is known that the results of corrosion rates measured with electrochemical techniques are generalized to the entire steel surface area exposed. That is also true for EIS measurements, so the results of this study were therefore averaged to the entire surface of the exposed steel bar, i.e., 17 cm^2^. When considering only the anodic areas, local corrosion rates can be much higher. For this reason, corrosion damage was visually assessed at the end of the exposure, thus verifying the electrochemical values and necessary information on the type of corrosion in order to interpret the electrochemical results. In order to obtain numerical information about the depths and volumes of the most severe local damage, μXCT scans were performed on each steel reinforcing bar after they were extracted from the mortar specimens at the end of exposure (W17; Figure 6b,c, Figure 7b,c, and Figure 8b,c). The correlation between the average corrosion rates calculated from the total impedance values (|*Z*|_total_) over the entire exposure and μXCT measurements at the end of exposure was assessed (Table 3). Although numerical interpretation of EIS spectra is generally the most difficult in cases where localized corrosion is severe, in our study, the agreement was fairly good. The numerical calculation of corrosion rates from the EIS parameters gave rather relevant results (the same order of magnitude) compared to the μXCT scans. It seems, however, that the EIS measurements underestimated values at lower corrosion rates yet significantly overestimated values at higher corrosion rates. It should be noted that μXCT has a resolution limit of 18 μm, so any damage or general reduction in diameter below this value cannot be taken into account. In this case, the corrosion rates calculated could therefore be ±55 μm/year, which is a significant degree of uncertainty in the measurement, especially when the corrosion activity is low. The resolution of μXCT can be significantly improved by using smaller specimens [29], but in this case, the corrosion exposure conditions can be problematic.

It was observed that steel in different types of AAMs corroded at various corrosion rates and in different forms, which can be related to different microstructure of mortars and different chemistry of their pore solutions. EIS provided certain information about these corrosion processes, but the specific relationships between the electrochemical response and the nature and rate of these processes remain unclear. These uncertainties are, in many ways, similar to those in ordinary cementitious materials, where the exact correlation between the type and rate of corrosion is still under investigation. It was also shown that the corrosion rates in individual AAMs were not directly connected to either the compressive strength or porosity values of these mortars; however, a more systematic analysis of the mechanical and physical properties [40,50] should be performed in order to draw definite conclusions. Change in pH and increase in chlorides over time were not investigated, and the correlation of their exact values to corrosion propagation is therefore unknown. On the other hand, it seems that the composition of individual AAMs additionally affects the electrochemical response. One of the main open issues remaining is whether these electrochemical responses can be attributed wholly to the microstructure, which controls the migration of ions, water, and oxygen, or if they also influenced by additional specific electrochemical processes not directly related to steel corrosion [27]. The use of alternative monitoring techniques (different sensors) should therefore be considered for further research.

## 5. Conclusions

This paper presents a study of corrosion in steel exposed for 17 weeks to various alkali-activated mortars, based on either fly ash (FA8), metakaolin (MK2), or slag (S3a-661). Corrosion was studied by means of electrochemical impedance spectroscopy (EIS), visual assessment, scanning electron microscopy (SEM), and X-ray computed microtomography (μCT). The following conclusions can be made:

- It was shown that the shapes of the EIS spectra significantly differed between the various AAMs but that they also changed over time. The corrosion rates assessed from the absolute impedance values [Z] of the EIS spectra measured were generally in agreement with the corrosion rates estimated from the actual corrosion damage, as measured by μXCT.
- X-ray computed microtomography (μXCT) provided relevant information regarding the type of corrosion and the extent of corrosion damage in the different AAMs, which ranged from very dense, small pits (FA8) to large and deep areas of corrosion without any pits nearby (MK2) or very shallow areas of corrosion with only a few small pits (S3a-661). These specifics need to be considered when interpreting the electrochemical measurements.
- It was shown that the corrosion rates in the different AAMs were not explicitly related to the compressive strength or porosity values of the mortars.
- In specific cases, the corrosion rates assessed from the EIS spectra evidently overestimated the actual corrosion rates. It is not clear whether this discrepancy was due to unsuitable modelling of the spectra or due to the presence of additional specific electrochemical processes not directly related to the corrosion of steel.

It is evident that a number of open questions related to the corrosion of steel in AAMs still remain. In order to investigate these issues, our future research will consider the use of alternative monitoring techniques not based on electrochemical modelling that enable differentiation between the various types of corrosion, i.e., physical monitoring methods (e.g., electrical resistance sensors) and other techniques (e.g., measurements of coupled currents with multi-electrode array sensors).

## Figures and Tables

**Table 1:** Mortar-mix designs used in the study.

Mortar/[g]	FA8	MK2	S3a-661
Fly ash (V-378/14)	455.9	-	-
Slag (V-138/15)	-	-	557.4
Metakaolin (V-63/15)	-	450.0	-
Water glass (V-25/15)	168.5	-	22.4
Water glass (V-502/14)	-	372.0	-
NaOH (V-44/15)	-	37.8	33.4
NaOH solution 41.7% (wt.) NaOH + 58.3% (wt.) H_2_O	64.4	-	-
Tap water	17.7	5.0	232.3
CEN Standard sand (EN 196-1)	1350.0	1350.0	1350.0
Na_2_O equivalent of precursors [%]	2.24	0.12	0.49

**Table 2:** Total impedance (|*Z*|_total_) values measured by EIS and calculated corrosion rates (*ν*_corr_) in the three alkali-activated mortars tested.

AAM Solution Type	|*Z*|_total_ [kΩ·cm^2^]			*ν*_corr_ [μm/year]		
	Week 1	Week 8	Week 17	Week 1	Week 8	Week 17
FA8	26	1.1	0.2	12	270	1510
MK2	8	2.2	0.9	38	140	340
S3a-661	117	19	39	3	16	8

**Table 3:** (**a**) Corrosion rates (*ν*_corr_) calculated from corrosion depths measured by μXCT and (**b**) average corrosion rates calculated from total impedance (|*Z*|_total_) values measured in three alkali-activated mortars after 17 weeks of exposure.

AAM Mortar Type	(a) μXCT Analysis				(b) EIS Measurements
	Corrosion Depths [μm]		Corrosion Rates, *ν*_corr_ [μm/year]		Average Corrosion Rates, *ν*_corr-EIS_ [μm/year]
	Average *d*_A_	Max *d*_MAX_	Average, *ν*_corr-A_	Max, *ν*_corr-MAX_	
FA8	38	225	117	690	590
MK2	49	375	150	1150	185
S3a-661	17	200	54	613	15

## Data Availability

The data presented in this study are available on request from the corresponding author.
